# Ceftazidime-Avibactam as Early Empiric Therapy for Gram-Negative Nosocomial Infections: A Risk-Stratified Empiric Use Informed by National AMR Surveillance Data 2024

**DOI:** 10.3390/antibiotics15070693

**Published:** 2026-07-16

**Authors:** Ahmad Subhi, Ruby Subhi, Yousef Sattar, Salma Alshamsi, Najiba Abdulrazzaq, Pramod Chhabrani

**Affiliations:** 1 Adult Infectious Disease-Department of Medicine, Al Qassimi Hospital, Emirates Health Services (EHS), Sharjah P.O. Box 3500, United Arab Emirates; so.alshamsi@ehs.gov.ae; 2 College of Medicine, Gulf Medical University, Ajman P.O. Box 4184, United Arab Emirates; 2023md12@mygmu.ac.ae (R.S.); 2023pcs04@mygmu.ac.ae (Y.S.); 3 Department of Medicine, Al Kuwait Hospital, Emirates Health Services (EHS), Dubai P.O. Box 22241, United Arab Emirates; najiba.abdulrazzaq@ehs.gov.ae (N.A.); pramod.chhabrani@ehs.gov.ae (P.C.)

**Keywords:** ceftazidime-avibactam, empiric therapy, carbapenem-resistant Enterobacterales, antimicrobial stewardship, UAE, nosocomial pneumonia, OXA-48, NDM, CAZ-AVI timing

## Abstract

Background: International evidence supports early initiation of ceftazidime-avibactam (CAZ-AVI) in carbapenem-resistant Enterobacterales (CRE) infections, with superior outcomes when therapy is initiated within 72 h. The applicability of this principle to the United Arab Emirates (UAE) requires contextualization against local antimicrobial resistance epidemiology. Methods: This consensus narrative review integrates two evidence streams: (1) national AMR surveillance data from the UAE National Antimicrobial Resistance Surveillance Report 2024—encompassing 195,108 non-duplicate isolates from 318 surveillance sites across all seven Emirates—providing phenotypic susceptibility and resistance rates for key gram-negative pathogens; and (2) a structured review of the published international evidence base on CAZ-AVI clinical efficacy, outcome data, and timing, identified through PubMed searches using the terms “ceftazidime-avibactam”, “empiric therapy”, “carbapenem-resistant Enterobacterales”, and “nosocomial infections”. The risk-stratification framework was developed through expert consensus by the authoring group, informed by the integrated evidence synthesis and structured around established clinical risk factors for carbapenem-resistant gram-negative infection. Results: National carbapenem resistance among Enterobacterales remains low (imipenem 3.4%R; meropenem 1.4%R), with *Klebsiella pneumoniae* exhibiting the highest carbapenem non-susceptibility (imipenem 5.8% NS; meropenem 2.8% NS). Direct CAZ-AVI susceptibility testing data were available only for *Pseudomonas aeruginosa* at the emirate level, showing consistently high susceptibility (88–93% across Abu Dhabi, Dubai, and the Northern Emirates); dedicated CAZ-AVI susceptibility data for *Klebsiella pneumoniae* and *Escherichia coli* were not reported in any emirate-level antibiogram, representing a significant national surveillance gap. The absence of carbapenemase genotype data (OXA-48 versus NDM/MBL) in the national surveillance dataset is identified as a further critical gap. Conclusions: CAZ-AVI is not supported as a universal empiric therapy for all nosocomial pneumonia in the UAE. A risk-stratified approach is proposed: standard anti-pseudomonal beta-lactams for general nosocomial infections, and early CAZ-AVI for pre-defined high-risk CRE-suspected patients, where the timing benefit is operationally justified primarily by the international outcome evidence, given that UAE-specific CAZ-AVI phenotypic data are currently available only for P. aeruginosa. Investment in carbapenemase molecular surveillance and expanded CAZ-AVI susceptibility reporting is recommended as a national priority to enable precise empiric prescribing.

## 1. Introduction

Antimicrobial resistance (AMR) represents one of the most consequential threats to global healthcare systems, with a projected 39.1 million deaths attributable to AMR between 2025 and 2050 under reference-scenario forecasts [1]. In the United Arab Emirates, AMR is recognized at the highest levels of public health governance, with a national surveillance system established in 2015 by the Ministry of Health and Prevention (MOHAP) in collaboration with the Dubai Health Authority, the Department of Health Abu Dhabi, and the Abu Dhabi Public Health Center [1].

Ceftazidime-avibactam (CAZ-AVI) is a combination beta-lactam/beta-lactamase inhibitor comprising ceftazidime, a third-generation cephalosporin, and avibactam, a non-beta-lactam beta-lactamase inhibitor [2,3]. Avibactam inhibits Ambler class A (including KPC and extended-spectrum beta-lactamases), class C (AmpC), and class D (OXA-48 and related enzymes) beta-lactamases, while providing no inhibitory activity against Ambler class B metallo-beta-lactamases (MBLs), including NDM-type carbapenemases [2,3]. This mechanistic profile defines both the therapeutic utility and the critical limitations of CAZ-AVI.

Multiple real-world studies from India, a healthcare geography with epidemiological relevance to the UAE given substantial population linkages, have shown the effectiveness of CAZ-AVI in gram-negative nosocomial infections, particularly hospital-acquired pneumonia (HAP) and ventilator-associated pneumonia (VAP). A systematic review and meta-analysis by Raman et al. established that early and appropriate antimicrobial therapy (AAT) versus inappropriate antimicrobial therapy (IAT) in gram-negative infections is associated with a lower unadjusted odds ratio for mortality (0.38; 95% CI 0.30–0.47) compared with IAT (unadjusted OR 2.66; 95% CI 2.12–3.35) [4]. Riccobene et al., in the largest real-world dataset to date examining newer antibacterials, including CAZ-AVI (229,320 ENT and 36,027 PsA susceptibility result records), demonstrated that delayed initiation in the fourth quartile (Q4, most delayed) versus the first quartile (Q1, earliest initiation) was associated with significantly increased hospital mortality and longer median post-culture length of stay [5].

The clinical evidence for early initiation is therefore well-established in the international literature. However, the critical and often under-examined variable is whether the selected empiric agent is actually active against the causative pathogen, a determination that, in the case of CRE, is totally dependent on the local carbapenemase gene epidemiology. The aim of this review is to critically evaluate whether CAZ-AVI is justified as early empiric therapy for gram-negative nosocomial infections in the UAE, by integrating UAE national AMR surveillance data with the international evidence base on CAZ-AVI efficacy and timing. We argue that the UAE 2024 National AMR Surveillance data, read carefully, supports a precise position: CAZ-AVI has a legitimate and evidence-based role as early empiric therapy in a pre-defined high-risk subgroup, but not across all nosocomial gram-negative infections.

## 2. Methods

This review was developed using a structured two-source evidence synthesis approach. First, national AMR surveillance data were extracted from the UAE National Antimicrobial Resistance Surveillance Report 2024 (MOHAP, published February 2026), the most comprehensive nationally representative microbiological dataset available for the UAE. Phenotypic susceptibility and resistance data were extracted for the primary gram-negative organisms of clinical interest: Klebsiella pneumoniae, Escherichia coli, Pseudomonas aeruginosa, and Acinetobacter baumannii. Both national aggregate and emirate-level antibiogram data were analyzed, with direct extraction of ceftazidime-avibactam (CAZ-AVI) susceptibility rates where available.

Second, a structured literature review was conducted using PubMed (last searched January 2026) to identify relevant clinical studies. Search terms included “ceftazidime-avibactam”, “empiric antibiotic therapy”, “carbapenem-resistant Enterobacterales”, “nosocomial pneumonia”, “hospital-acquired pneumonia”, “ventilator-associated pneumonia”, “antimicrobial stewardship”, and “antibiotic timing outcomes”. Studies were included if they reported clinical outcomes (mortality, length of stay, clinical cure) associated with CAZ-AVI use or early appropriate antibiotic therapy in gram-negative nosocomial infections. No formal PRISMA systematic review methodology was applied; this work is a narrative review and expert consensus position.

The risk-stratification framework presented in Table 1 was developed through expert consensus by the authoring group, comprising infectious disease physicians and clinical microbiologists from UAE healthcare facilities. The framework was structured around three validated clinical risk factors for carbapenem-resistant gram-negative infection: prior carbapenem exposure, prolonged ICU stay (defined as >7 days), and prior documented CRE or CRPA colonization. These risk factors were selected based on their consistent identification as independent predictors of carbapenem-resistant infection in published clinical risk-stratification literature. The framework is explicitly proposed as a consensus clinical tool to guide empiric prescribing decisions pending facility-level validation studies, which are identified as a research priority.

## 3. The UAE Antimicrobial Resistance Landscape: 2024 National Data

### 3.1. Surveillance System Overview

The UAE National AMR Surveillance Report 2024, published February 2026 by MOHAP, represents the most comprehensive nationally representative AMR dataset available for the UAE. The 2024 dataset encompasses 195,108 non-duplicate diagnostic isolates from 318 surveillance sites, 87 hospitals, and 231 ambulatory centers/clinics [1]. Served by 54 clinical microbiology laboratories across all seven Emirates, the system is highly representative of inpatients and ICU patients (57.6% of participating hospitals) and the public sector (100% participation). All participating laboratories follow CLSI guidelines for antimicrobial susceptibility testing; 98% are laboratory accredited by CAP or ISO-15189 [1].

For the purposes of this analysis, the primary organisms of interest are *Klebsiella pneumoniae* and *Escherichia coli* (the dominant Enterobacterales causing nosocomial infections) and *Pseudomonas aeruginosa* (which contributes significantly to a broad range of healthcare-associated infections and is a particularly frequent causative pathogen in hospital-acquired and ventilator-associated pneumonia). The report provides both national and emirate-level antibiograms, including direct CAZ-AVI susceptibility data.

### 3.2. Carbapenem Resistance: National Picture

The headline finding is that carbapenem resistance among Enterobacterales remains relatively low in the UAE [1]. Table 2 summarizes the national carbapenem resistance data from the 2024 report.

These figures have important implications for the empiric prescribing strategy. The low overall carbapenem resistance rate means that the absolute number of CRE infections in any given institution is small. Standard anti-pseudomonal beta-lactam regimens, such as piperacillin-tazobactam, cefepime, or carbapenems, remain appropriately active against the overwhelming majority of nosocomial gram-negative pathogens in the UAE. Carbapenems retain 97–99% susceptibility against *E. coli* and 94–97% against *K. pneumoniae* nationally [1].

*Pseudomonas aeruginosa* presents a distinct profile. With imipenem resistance of 13.5% and meropenem resistance of 9.7%, carbapenem-resistant *P. aeruginosa* (CRPA) is a proportionally more common clinical problem in the UAE than CRE [1]. This distinction is clinically relevant for CAZ-AVI positioning, as discussed below.

The national report also identifies a concerning resistance pattern: MDR rates are increasing for both *E. coli* (↑) and *K. pneumoniae* (↑), reaching 47.7% and 29.7%, respectively, in 2024 [1,6]. This upward trend underscores the importance of preserving the activity of newer agents through stewardship-guided, risk-stratified use rather than empiric overuse.

From a stewardship program perspective, ertapenem resistance in *K. pneumoniae* deserves specific attention as an early screening marker for CRE. Ertapenem, unlike imipenem or meropenem, lacks intrinsic activity against *Pseudomonas aeruginosa* and has a narrower spectrum limited to Enterobacterales. Within this group, ertapenem non-susceptibility in *K. pneumoniae* can serve as an early phenotypic signal of carbapenemase production, particularly KPC or OXA-48-like enzymes, as these enzymes may hydrolyze ertapenem preferentially or produce non-susceptibility at lower enzyme expression levels before imipenem or meropenem MICs rise above the resistance breakpoint. In the national dataset, the *K. pneumoniae* ertapenem %R of 3.0% (Table 2) exceeds its meropenem %R of 2.6%, a pattern consistent with early carbapenemase emergence. Antimicrobial stewardship programs in UAE hospitals should incorporate routine ertapenem testing of *K. pneumoniae* isolates and flag ertapenem non-susceptibility as a trigger for confirmatory carbapenemase testing, CRE isolation precautions, and prospective review of empiric therapy appropriateness.

### 3.3. CAZ-AVI Susceptibility: Emirate-Level Antibiogram Data

The 2024 report provides direct CAZ-AVI (ceftazidime/avibactam) susceptibility data in emirate-level cumulative antibiograms, but only for some of the priority gram-negative pathogens [1]. Table 3 summarizes the available CAZ-AVI susceptibility data and highlights an important gap in organism-level reporting.

Ceftazidime-avibactam susceptibility is not separately reported for *K. pneumoniae* or *E. coli* in any of the three emirate-level antibiograms; only ceftazidime-alone and carbapenem susceptibility are available for these organisms. Because *K. pneumoniae* is the predominant cause of CRE in the UAE (Section 3.2), this is the single most consequential gap identified in this review: the organism for which empiric CAZ-AVI decision-making matters most is also the organism for which the national surveillance system currently provides no direct phenotypic evidence of CAZ-AVI activity. Closing this gap should be considered a priority for future antibiogram cycles (Section 6.2).

The high CAZ-AVI susceptibility in *P. aeruginosa* is consistent across all three emirate-level antibiograms (88% Northern Emirates, 90% Abu Dhabi, 93% Dubai) [1]. Given that carbapenem-resistant *P. aeruginosa* represents a proportionally larger share of the resistant nosocomial pathogen burden in the UAE than CRE, and that it is the only organism for which the national surveillance system provides direct CAZ-AVI phenotypic data, this finding provides the strongest—and currently only—UAE-specific phenotypic support for CAZ-AVI’s empiric role, particularly in ventilator-associated pneumonia risk scenarios.

### 3.4. The Critical Surveillance Gap: Absence of Carbapenemase Genotype Data

The most significant limitation of the UAE 2024 National AMR Report for the purposes of this analysis is the absence of carbapenemase gene typing data. The report is entirely phenotypic; it categorizes isolates as susceptible, intermediate, or resistant based on minimum inhibitory concentration or inhibition zone diameter breakpoints but does not characterize the underlying resistance mechanism [1].

This gap is not a criticism of the surveillance system, which is appropriately designed for epidemiological monitoring, but it has direct consequences for empiric prescribing decisions involving CAZ-AVI. The central mechanistic distinction is:

- OXA-48 and OXA-48-like carbapenemases (Ambler class D serine enzymes): inhibited by avibactam. CAZ-AVI is clinically active [2,3].
- Kpc Carbapenemases (Ambler Class A Serine Enzymes): inhibited by Avibactam. CAZ-AVI is clinically active [2,3].
- NDM, VIM, IMP and other metallo-beta-lactamases (Ambler class B, requiring zinc): NOT inhibited by avibactam. CAZ-AVI has no clinically relevant activity [2,3].

Phenotypic CAZ-AVI susceptibility data, where available, reflect the net clinical activity of CAZ-AVI against all tested isolates, regardless of mechanism. For example, even if a future antibiogram cycle reports a dedicated CAZ-AVI susceptibility figure for *K. pneumoniae*, that figure alone could not distinguish a resistant fraction driven by OXA-48 (against which CAZ-AVI is active) from one driven by NDM carriage (against which it is not). These two scenarios would require entirely different empiric strategies and have profoundly different implications for CAZ-AVI’s reliability as a monotherapy agent—underscoring that closing the current CAZ-AVI reporting gap for *K. pneumoniae* would still need to be paired with carbapenemase genotyping to be clinically actionable.

The report notes the absence of a UAE national reference laboratory for AMR (NRL-AMR), which would typically be the institution responsible for confirmatory testing and molecular epidemiology [1]. Establishing this capacity and incorporating carbapenemase gene typing into the national surveillance protocol is identified in this paper as a priority recommendation.

The limitations of relying on phenotypic surveillance data alone for empiric prescribing decisions of a mechanistically selective agent such as CAZ-AVI warrant explicit discussion. Five key limitations are identified. First, where phenotypic CAZ-AVI susceptibility data are available currently only for *P. aeruginosa,* they reflect the aggregate activity of CAZ-AVI across all tested isolates, regardless of resistance mechanism; for example, the 93% susceptibility rate in Dubai *P. aeruginosa* is pharmacologically uninformative on its own without knowing whether the small resistant fraction is mediated by mechanisms avibactam can overcome (e.g., AmpC overexpression, porin loss) or those it cannot (e.g., VIM/IMP metallo-beta-lactamases). For *K. pneumoniae*, the principal CRE pathogen in the UAE, this ambiguity cannot even be assessed at present, since no emirate-level antibiogram reports a dedicated CAZ-AVI susceptibility figure for this organism at all. Second, the national antibiogram data are derived from all clinical isolates, not from confirmed nosocomial or ICU-acquired infections specifically; carbapenem resistance prevalence may be higher in the subset of organisms acquired in high-acuity settings, meaning that the national figures likely underestimate the true CRE burden in the patient population most likely to receive empiric CAZ-AVI. Third, the UAE surveillance system captures microbiological data from participating hospitals and ambulatory sites but does not link to outcomes data (mortality, clinical cure), meaning it is not possible to directly calculate the clinical impact of prescribing patterns from the surveillance dataset alone. Fourth, interoperability between different surveillance systems and participating laboratories may introduce variability in breakpoint application and minimum inhibitory concentration testing methodology, which could affect the comparability of susceptibility data across the Emirates. Fifth, the 2024 national report represents a single annual cross-section; temporal trends in resistance gene distribution, which may shift as clonal lineages spread or new resistance determinants are imported, cannot be captured from a single year’s data. These limitations collectively reinforce the conservative empiric position advocated in this review: phenotypic data can support a risk-stratified empiric framework but cannot substitute for molecular characterization in guiding high-stakes prescribing decisions involving mechanistically selective agents.

## 4. International Evidence on CAZ-AVI Timing and Outcomes

### 4.1. The Principle of Early Appropriate Antimicrobial Therapy

The prognostic importance of early appropriate antimicrobial therapy (AAT) in serious gram-negative infections is one of the most consistent findings in the infectious disease literature. The seminal systematic review by Gowri Raman [4] included comprehensively 57 studies of hospitalized patients with gram-negative infections, quantifying this association with precision: AAT was associated with a lower unadjusted odds ratio for mortality of 0.38 (95% CI 0.30–0.47) compared with inappropriate antimicrobial therapy (IAT) (unadjusted OR 2.66; 95% CI 2.12–3.35). IAT was further associated with longer hospital stays and higher healthcare costs [3].

A retrospective cohort study by Sarah E. Battle [7] of 830 adults with gram-negative bloodstream infections demonstrated that inappropriate empirical antimicrobial therapy was associated with prolonged hospital length of stay (LOS) in a Kaplan–Meier analysis (log-rank *p* = 0.004). The median hospital LOS was significantly lower in patients receiving appropriate therapy in both good (Bloodstream Infection Mortality Risk Score [BSIMRS] < 5) and guarded (BSIMRS ≥ 5) prognosis groups. Taken together, Raman et al. and Battle et al. address complementary endpoints, mortality and LOS, respectively and collectively establish that both survival and resource utilization are meaningfully impacted by antibiotic appropriateness. Where Raman et al. is limited by the heterogeneity inherent in pooled meta-analytic data across different infection types, Battle et al. provides granularity through survival analysis in a single infection type. The consistent direction of effect across these methodologically distinct studies strengthens the evidence that early appropriate therapy is a clinically meaningful target [7].

### 4.2. Real-World Evidence for CAZ-AVI

Two Indian real-world studies are directly relevant to the UAE clinical context, given the substantial overlap in patient demographics, travel patterns, and healthcare utilization between the two countries.

Neha Gupta examined the real-world effectiveness of CAZ-AVI in a cohort of Indian patients with pneumonia (HAP/VAP) caused specifically by carbapenem-resistant *Klebsiella pneumoniae* [8]. The study, conducted across multiple sites, including the Fortis Memorial Research Institute (Gurugram), SL Raheja Hospital (Mumbai), and Fortis Hospital (Kolkata), showed clinical cure rates supporting CAZ-AVI’s role in HAP/VAP caused by carbapenem-resistant organisms. A key strength of this study is its multi-site design capturing geographic heterogeneity; however, its retrospective methodology and absence of molecular carbapenemase typing limit conclusions about optimal patient selection criteria [8]. Subhash Todi, in a multi-center retrospective analysis across nine advanced care centers in India, including Apollo Hospitals Chennai, Gleneagles Global Hospitals, and Deenanath Mangeshkar Hospital Pune, provided wider real-world evidence on CAZ-AVI effectiveness across multiple gram-negative infection types, including carbapenem-resistant *K. pneumoniae*, *P. aeruginosa*, and Enterobacterales. Compared with Neha Gupta publication, the Todi dataset offers broader coverage of infection types and organism classes, lending greater generalizability, though it shares the same limitations of a retrospective design and heterogeneous treatment protocols across sites [9]. Both studies are cited here as evidence of CAZ-AVI’s clinical effectiveness in HAP/VAP caused by carbapenem-resistant gram-negative organisms in a demographically overlapping patient population; however, a critical epidemiological qualification must be applied before extrapolating their findings to the UAE. India is a hyperendemic region for metallo-beta-lactamase (MBL)-producing organisms, with NDM-type carbapenemases predominating among CRE isolates. If this resistance gene distribution were directly applicable to the UAE, empiric CAZ-AVI monotherapy would be expected to fail in a substantial proportion of CRE cases, since avibactam has no inhibitory activity against class B MBLs. The clonal distribution and resistance gene epidemiology of the Gulf region may differ substantially from that of South Asia. Accordingly, these Indian studies should be read as providing evidence for the general clinical benefit of appropriately targeted CAZ-AVI therapy, not as validating empiric monotherapy in a setting where MBL prevalence is unknown. This distinction further reinforces the central recommendation of this review: that carbapenemase molecular surveillance is a national priority in the UAE before broader empiric CAZ-AVI use can be safely recommended [1].

### 4.3. The Timing Imperative

The most statistically robust contemporary evidence on CAZ-AVI timing comes from Todd Riccobene, who provided the first real-world evidence comparing early versus delayed use of newer antibacterials, including CAZ-AVI, with 229,320 Enterobacterales and 36,027 *Pseudomonas aeruginosa* susceptibility result records, with CAZ-AVI being the most frequently prescribed newer antibacterial. The study stratified patients into quartiles (Q1 through Q4) based on time from culture result to initiation of newer antibacterial therapy, where Q1 represents the earliest initiators and Q4 the most delayed. The study demonstrated that delayed initiation of newer antibacterials was associated with significantly increased hospital mortality, with higher odds ratios in Q4 versus Q1 for both ENT (OR 1.96; 95% CI 1.13–3.42; *p* = 0.0182) and PsA (OR 1.84; 95% CI 1.02–3.32; *p* = 0.0423). Delayed initiation was further linked to significantly longer median post-culture length of stay in Q4 versus Q1 for both organisms (35.4 vs. 11.1 days for ENT, *p* < 0.0001; 30.9 vs. 12.5 days for PsA, *p* < 0.0001) [6].

The consistent message across this evidence base is that patients who began therapy within 72 h of infection onset consistently achieved better outcomes than those treated after 72 h [6,10]. However, a slight difference applies to the interpretation of all these studies in the UAE context: the outcome benefit of early therapy is entirely contingent on it being appropriately active against the causative organism. Early inappropriate therapy is not equivalent to early appropriate therapy, and in settings with high MBL prevalence, early CAZ-AVI monotherapy may constitute early inappropriate therapy for a substantial proportion of CRE episodes.

## 5. Synthesis: UAE-Contextualized Position on CAZ-AVI Empiric Use

### 5.1. What the Data Do and Do Not Support

A precise reading of the UAE 2024 AMR data, integrated with the international evidence base, permits the following conclusions:

The data do not support universal empiric CAZ-AVI for all gram-negative nosocomial infections. The national carbapenem susceptibility of 97–99% for meropenem against *E. coli* and 97% against *K. pneumoniae* renders CAZ-AVI unnecessary and stewardship-harmful as a default empiric choice [1]. Bringing a last-resort agent into effective action in settings where standard carbapenems retain near-complete activity would accelerate resistance without clinical benefit.

The data support risk-stratified early CAZ-AVI for pre-defined high-risk patients. The phenotypic susceptibility data demonstrate 88–93% CAZ-AVI activity in *P. aeruginosa* across all three emirates [1]; dedicated CAZ-AVI susceptibility data for *K. pneumoniae* are not available nationally, so the case for early empiric use in suspected CRE rests primarily on international timing evidence and mechanistic plausibility rather than UAE-specific phenotypic confirmation. This evidence supports early empiric use in patients where CRE or carbapenem-resistant *P. aeruginosa* is genuinely likely, and where waiting for culture susceptibility results would incur the mortality and LOS penalties documented in the international literature [4,7].

The data identify a critical gap in the absence of CAZ-AVI susceptibility reporting and carbapenemase molecular surveillance for *K. pneumoniae*. No emirate-level antibiogram reports a dedicated CAZ-AVI susceptibility figure for *K. pneumoniae*, meaning there is currently no UAE phenotypic evidence base for CAZ-AVI activity against the organism most responsible for CRE. Even if such data were reported in future cycles, their interpretability would still be limited by the absence of carbapenemase genotyping: a hypothetical CAZ-AVI susceptibility figure for this organism could not be interpreted without knowing whether the resistant fraction is driven by OXA-48 (against which CAZ-AVI remains active) or NDM/MBL (against which empiric CAZ-AVI monotherapy would fail in a clinically unacceptable proportion of cases). Both gaps should be closed before empiric CAZ-AVI use in suspected CRE can be supported by UAE-specific evidence rather than international data alone.

### 5.2. Proposed Risk-Stratified Empiric Framework

Based on the integrated analysis of UAE national AMR data and international evidence, the following empiric strategy is proposed for nosocomial gram-negative infections in the UAE. The framework draws on risk factors that have been consistently validated as independent predictors of carbapenem-resistant infection in the published literature. Prior carbapenem exposure has been identified as a significant risk factor for CRE in multiple multivariate analyses, with odds ratios ranging from 2.1 to 6.4 across studies. Prolonged ICU stay (>7 days) and prior CRE colonization are similarly well-established predictors incorporated into existing risk scores such as the INCREMENT-CPE score and the Tumbarello risk score for carbapenemase-producing Klebsiella pneumoniae. The three-tier structure (standard risk, high CRE risk, CRPA risk) reflects the distinct epidemiological profiles of these pathogen groups in the UAE national data, where CRPA (imipenem 13.5%R) represents a proportionally higher carbapenem resistance prevalence than CRE (imipenem 3.4%R). It is important to note that this framework has not been prospectively validated in a UAE clinical cohort; this is identified as a research priority. Until local validation data are available, the framework should be applied as a clinical decision-support tool subject to individualized clinical judgment and antimicrobial stewardship team review, rather than as a rigid protocol.

### 5.3. The De-Escalation Imperative

Risk-stratified empiric prescribing is only stewardship-sound when paired with systematic de-escalation. Early CAZ-AVI use in high-risk patients must be accompanied by a commitment to de-escalate to the narrowest active agent once culture and susceptibility results are available, typically within 48–72 h. For OXA-48-producing CRE confirmed on molecular testing, CAZ-AVI monotherapy remains appropriate. For NDM-producing isolates, CAZ-AVI should be discontinued, and an MBL-active regimen should be used instead. For susceptible organisms, de-escalation to standard carbapenems or even narrower agents is mandated. When MBL-producing organisms are suspected or confirmed, the CAZ-AVI + aztreonam combination represents a well-established and pharmacologically rational strategy. The rationale is as follows: aztreonam, a monobactam, is intrinsically stable against hydrolysis by metallo-beta-lactamases (class B enzymes, including NDM, VIM, and IMP), making it one of the few beta-lactams that retains activity against MBL-producing organisms. However, MBL-producing strains frequently co-produce ESBLs or AmpC enzymes (class A or class C), which would otherwise rapidly destroy aztreonam and render it ineffective as monotherapy. Avibactam, by inhibiting these co-produced class A and class C enzymes, protects aztreonam from hydrolysis and restores its antimicrobial activity. The combination thus achieves coverage of MBL-producing CRE that neither agent provides alone, making it the preferred combination strategy in Tier 4 patients pending molecular characterization of the resistance mechanism.

Failure to de-escalate systematically eliminates the stewardship rationale for risk-stratified empiric prescribing and accelerates the selection of CAZ-AVI resistance, a consequence that would undermine the very outcome that the strategy is designed to preserve.

## 6. Recommendations

### 6.1. Clinical Recommendations

Based on the analysis presented, the following clinical recommendations are proposed for UAE healthcare facilities:

- Implement risk-stratified empiric prescribing for nosocomial gram-negative infections, as outlined in Table 1, replacing blanket empiric escalation strategies.
- Restrict empiric CAZ-AVI to Tier 2 (high CRE risk) and Tier 3 (CRPA risk) patients with pre-defined, documented risk factors, reviewed on a per-case basis by the antimicrobial stewardship team.
- Mandate systematic de-escalation within 48–72 h of culture and susceptibility results for all patients commenced on empiric CAZ-AVI.
- In facilities where CRPA is the primary carbapenem-resistant pathogen risk (consistent with UAE national data showing higher *P. aeruginosa* than Enterobacterales carbapenem resistance), CAZ-AVI should be considered alongside ceftolozane-tazobactam as the preferred empiric anti-CRPA agent, selected based on facility-specific antibiogram data.
- For patients with epidemiological risk factors for NDM or other MBL-producing organisms, empiric CAZ-AVI monotherapy may not be sufficient. CAZ-AVI combined with aztreonam, or empiric cefiderocol where available, should be considered pending molecular characterization.

### 6.2. Surveillance Recommendations

The following surveillance enhancements are recommended as national priorities:

- Establish a national reference laboratory for antimicrobial resistance (NRL-AMR) with the ability to perform molecular testing, as this was identified as a gap in the 2024 national report [1].
- Incorporate mandatory carbapenemase gene typing (at minimum OXA-48, KPC, NDM, VIM, IMP) for all CRE isolates at sentinel surveillance sites.
- Segregate CAZ-AVI susceptibility data in future national antibiograms based on carbapenem resistance (CRE vs. non-CRE), as this provides more clinically useful information than overall CAZ-AVI susceptibility rates.
- Incorporate a dedicated ceftazidime-avibactam (CAZ-AVI) susceptibility column for *K. pneumoniae*, *E. coli*, and *A. baumannii* in future national antibiogram cycles; currently, only *P. aeruginosa* has CAZ-AVI susceptibility separately reported across all three emirate-level antibiograms, leaving a significant reporting gap for *K. pneumoniae*, the organism most responsible for CRE in the UAE [1].
- Strengthen reporting completeness from the Northern Emirates and from private sector ambulatory facilities, where current representation remains limited.

### 6.3. Stewardship Program Recommendations

- Healthcare facilities managing ICU patients should develop facility-level CAZ-AVI prescribing protocols incorporating the risk-stratification framework and de-escalation requirements.
- Point-of-care rapid molecular diagnostics for carbapenemase gene identification should be evaluated and introduced as a priority investment, enabling same-day empiric therapy optimization, rather than awaiting phenotypic susceptibility results.
- Regular audit of CAZ-AVI and other novel beta-lactam beta-lactamase inhibitor prescribing appropriateness, capturing indication, timing, and de-escalation compliance should be incorporated into existing antimicrobial stewardship program metrics.

## 7. Conclusions

The international evidence base supporting early initiation of CAZ-AVI in CRE and carbapenem-resistant gram-negative infections is strong and consistent. Improved outcomes are seen when appropriate therapy is started within 72 h, while delays are associated with significantly higher mortality odds ratios and prolonged hospital length of stay [4,7]. This principle remains clinically relevant and applicable to the UAE.

The UAE 2024 National AMR Surveillance Report provides a robust evidence base for carbapenem resistance epidemiology, though direct CAZ-AVI phenotypic data are more limited than initially apparent. Carbapenem resistance remains nationally low among Enterobacterales, preserving the clinical utility of standard agents for the majority of nosocomial infections. Direct CAZ-AVI susceptibility data are reported only for *P. aeruginosa*, demonstrating strong and consistent activity (88–93% across Abu Dhabi, Dubai, and the Northern Emirates); dedicated CAZ-AVI susceptibility data for *K. pneumoniae*, the principal CRE pathogen in the UAE, are not currently reported in any emirate-level antibiogram, and this surveillance gap should be prioritized in future reporting cycles [1].

What the national data cannot support and what no phenotypic surveillance system alone can provide is the gene-level precision needed to make CAZ-AVI monotherapy a safe, universal empiric choice for CRE. The absence of carbapenemase molecular epidemiology data from the national surveillance system is the critical gap that limits the strength of the empiric recommendation [1].

The clinically and stewardship-sound position, integrating UAE national surveillance data with international outcome evidence, is as follows: ceftazidime-avibactam (CAZ-AVI) should be recommended as early empiric therapy only within a clearly defined, risk-stratified high-risk subgroup, rather than as routine empiric therapy for all nosocomial gram-negative infections. In patients with true risk factors for carbapenem-resistant Enterobacterales (CRE) or carbapenem-resistant *Pseudomonas aeruginosa* (CRPA), the urgency of timely effective therapy supports early initiation. In contrast, for patients without these risk factors, national data indicate that standard beta-lactam regimens remain adequately active and are more appropriate from a stewardship perspective.

The highest clinical priority for improving the precision of this recommendation is investment in national carbapenemase molecular surveillance—a capability that would transform the UAE’s ability to move from phenotypic to mechanism-informed empiric prescribing, and from consensus expert opinion to data-driven individual patient decisions.

## Figures and Tables

**Table 1:** Proposed risk-stratified empiric therapy framework for nosocomial gram-negative infections, UAE.

Risk Tier	Patient Profile	Empiric Recommendation	Evidence Basis
Tier 1 (Standard Risk)	No prior carbapenem exposure; no prolonged ICU stay; no prior CRE colonization	Anti-pseudomonal beta-lactam ± aminoglycoside per local antibiogram	UAE 2024: carbapenem susceptibility 94–99% in key pathogens
Tier 2 (High-Risk CRE)	Prior carbapenem exposure; prolonged ICU stay (>7 days); prior CRE colonization; HAP/VAP with deterioration on carbapenem	Early CAZ-AVI (within 72 h); de-escalate on culture results	International timing evidence supports ≤ 72 h initiation; UAE-specific CAZ-AVI susceptibility data for *K. pneumoniae* are not available (national surveillance gap)
Tier 3 (CRPA Risk)	Ventilator-associated pneumonia; structural lung disease; prior *P. aeruginosa* isolation; immunocompromised	Early CAZ-AVI or ceftolozane-tazobactam (per local *P. aeruginosa* antibiogram)	UAE 2024: *P. aeruginosa* CAZ-AVI 88–93%S across all three emirates; the only UAE organism with confirmed CAZ-AVI phenotypic data
Tier 4 (MBL Concern)	Epidemiological risk for NDM/MBL-producing organism (recent travel to endemic region; institutional outbreak)	CAZ-AVI + aztreonam combination pending molecular result; or cefiderocol, if available	Mechanistic: avibactam does not cover MBL. Aztreonam stable to MBL; avibactam protects aztreonam from ESBL/AmpC

CAZ-AVI = ceftazidime-avibactam; CRE = carbapenem-resistant Enterobacterales; CRPA = carbapenem-resistant Pseudomonas aeruginosa; MBL = metallo-beta-lactamase; HAP = hospital-acquired pneumonia; VAP = ventilator-associated pneumonia.

**Table 2:** Carbapenem non-susceptibility among key gram-negative pathogens, UAE 2024.

Organism	N (Isolates)	IPM %R	MEM %R	ETP %R
Enterobacterales (all)	93,139	3.4	1.4	—
*E. coli*	53,918	1.1	0.9	1.2
*K. pneumoniae*	21,594	3.9	2.6	3.0
*P. aeruginosa*	12,625	13.5	9.7	—
*Acinetobacter baumannii*	2546	5.6	5.9	—

For all organisms in this table, the %R value represents resistant isolates only. The overall non-susceptibility rate may be higher because it includes both resistant (R) and intermediate (I) isolates. These values are reported separately to align with CLSI breakpoint reporting conventions. For *Acinetobacter baumannii*, the identical IPM and MEM %R values reflect the national surveillance dataset as reported. This resistance rate is notably lower than that reported in many international nosocomial series and may reflect the composition of the UAE surveillance sample, which includes both hospital and ambulatory isolates. Carbapenem-resistant *A. baumannii* is not a primary target for CAZ-AVI because avibactam lacks activity against the OXA-23-like carbapenemases and other OXA-type enzymes that typically mediate carbapenem resistance in *A. baumannii* (see Methods section). ETP = ertapenem; dashes (—) indicate that ertapenem is not routinely tested or reported for non-fermenters (*P. aeruginosa*, *A. baumannii*) in the national surveillance protocol, as ertapenem has no reliable activity against these organisms. IPM = imipenem; MEM = meropenem. Data source: UAE National AMR Surveillance Report 2024, Tables 4.4.1.1, 4.4.2.1, and 4.4.4.1 [1].

**Table 3:** Ceftazidime-avibactam (CAZ-AVI) susceptibility by organism and emirate, UAE 2024.

Organism	Emirate	N	CAZ-AVI %S	Clinical Implication
*P. aeruginosa*	Abu Dhabi	6390	90%	Strong support for CAZ-AVI
*P. aeruginosa*	Dubai	3597	93%	Strong support for CAZ-AVI
*P. aeruginosa*	Northern Emirates	2635	88%	Strong support for CAZ-AVI

Data source: UAE National AMR Surveillance Report 2024, Tables 4.2.2.2 (Abu Dhabi), 4.2.3.2 (Dubai), and 4.2.4.2 (Northern Emirates) [1]. CAZ-AVI = ceftazidime-avibactam; %S = percent susceptible. *Pseudomonas aeruginosa* is the only organism among the four priority gram-negative pathogens reviewed for which a dedicated CAZ-AVI susceptibility column is reported in all three emirate-level antibiograms. Ceftazidime-avibactam susceptibility is not separately reported for *Klebsiella pneumoniae*, *Escherichia coli*, or *Acinetobacter baumannii* in any of the three antibiograms; only ceftazidime-alone and carbapenem susceptibility are available for these organisms. For *A. baumannii* specifically, this absence is mechanistically unsurprising, since avibactam has no inhibitory activity against the class D oxacillinases (OXA-23-like carbapenemases and other OXA-type enzymes) that predominantly drive *A. baumannii* carbapenem resistance in the UAE, and CAZ-AVI has no established clinical indication for *A. baumannii* infections. For *K. pneumoniae* and *E. coli*, however, the absence of dedicated CAZ-AVI reporting is a genuine surveillance gap, since these organisms—particularly K. pneumoniae—are the principal drivers of CRE in the UAE and are the organisms for which empiric CAZ-AVI decision-making is most clinically relevant.

## Data Availability

No new data were created in this study. Data supporting the findings are derived from the publicly available UAE National Antimicrobial Resistance Surveillance Report 2024, published by the Ministry of Health and Prevention (MOHAP), and from previously published literature cited throughout the manuscript.
